# Expression of Concern: Extracellular regulated kinase 5 mediates osteoporosis through modulating viability and apoptosis of osteoblasts in ovariectomized rats

**DOI:** 10.1042/BSR20190432_EOC

**Published:** 2025-10-09

**Authors:** Tuan-Mao Guo, Yan-Li Xing, Hai-Yun Zhu, Lan Yang, Guo-Xiong Liu, Xi-Min Qiao

**Affiliations:** 1 Department of Orthopedics, Xianyang Central Hospital, Xianyang, P.R, 712000, China; 2 Department of Urinary Surgery, Xianyang Central Hospital, Xianyang, P.R, 712000, China

*Bioscience Reports* has been made aware of potential issues surrounding the scientific validity of this paper and hence is issuing an Expression of Concern to notify readers whilst the Editorial Office investigates. Irregularities have been noted in the flow cytometry graphs – the authors were contacted for comment but have not yet responded or provided requested raw data.

